# BIOADI: a machine learning approach to identifying abbreviations and definitions in biological literature

**DOI:** 10.1186/1471-2105-10-S15-S7

**Published:** 2009-12-03

**Authors:** Cheng-Ju Kuo, Maurice HT Ling, Kuan-Ting Lin, Chun-Nan Hsu

**Affiliations:** 1 Institute of Information Science, Academia Sinica, Taipei 115, Taiwan, Republic of China; 2 School of Chemical and Life Sciences, Singapore Polytechnic, Republic of Singapore; 3 Institute of Biomedical Informatics, National Yang-Ming University, Taipei 112, Taiwan, Republic of China; 4 Department of Zoology, The University of Melbourne, Parkville, Victoria, Australia

## Abstract

**Background:**

To automatically process large quantities of biological literature for knowledge discovery and information curation, text mining tools are becoming essential. Abbreviation recognition is related to NER and can be considered as a pair recognition task of a terminology and its corresponding abbreviation from free text. The successful identification of abbreviation and its corresponding definition is not only a prerequisite to index terms of text databases to produce articles of related interests, but also a building block to improve existing gene mention tagging and gene normalization tools.

**Results:**

Our approach to abbreviation recognition (AR) is based on machine-learning, which exploits a novel set of rich features to learn rules from training data. Tested on the AB3P corpus, our system demonstrated a F-score of 89.90% with 95.86% precision at 84.64% recall, higher than the result achieved by the existing best AR performance system. We also annotated a new corpus of 1200 PubMed abstracts which was derived from BioCreative II gene normalization corpus. On our annotated corpus, our system achieved a F-score of 86.20% with 93.52% precision at 79.95% recall, which also outperforms all tested systems.

**Conclusion:**

By applying our system to extract all short form-long form pairs from all available PubMed abstracts, we have constructed BIOADI. Mining BIOADI reveals many interesting trends of bio-medical research. Besides, we also provide an off-line AR software in the download section on http://bioagent.iis.sinica.edu.tw/BIOADI/.

## Background

Protein/gene name recognition (NR) [1,2], is one of the most challenging tasks in biomedical text mining [3]. Solving the problem of NR will allow for more complex text mining tasks to be addressed [4] as it is a prerequisite for information extraction and advanced text mining [3,5,6]. One of the main reasons of the challenging is high variation of terms that are not explicitly reflected in biomedical ontologies [7]. It is common that biological entities can have several names. For example, PTEN and MMAC1 refers to the same entity [8]. It was estimated that one-third of biological terms are variants [9].

A number of important studies in this area include GAPSCORE [10], which examines the appearance, morphology and context of named entities before applying a classifier trained using these features (59% precision and 50% recall). ABNER [11] employed a conditional random field model and achieved precisions between 58.2% to 85.4% and recall between 53.9% and 79.8% for different target entities. Other groups had attempted combinations of approaches to improve precision [12-16].

Abbreviation recognition (AR) is related to NR and can be considered as a pair recognition task of a terminology (may be a phrase or an entity) and its corresponding abbreviation from free text. In this manuscript, we denote "LF" to mean "the long form of the term" and "SF" to mean "the abbreviation or the short form of the term". Since the name of most protein and gene names are rather lengthy, most researchers tend to abbreviate their names in published manuscripts. As a result, AR can serve as a precursor of a number of applications. For example, building a term index of a text database to retrieve articles of related interests [17] or to link text-mined protein interaction networks [18-20]. Hence, it seems plausible to use AR as a first-pass in NER. In the simplest sense, AR may be used to assist term boundaries of entity names in free text, such as reported in [21,22].

AR is generally considered as a simpler problem than NER and had been shown by the performance of AR systems [8]. For example, Stanford University's Abbreviation Server [23,24] demonstrated 97% precision at 22% recall and 95% precision at 75% recall. AbbRE [25] and the system by Schwartz et al. [26] achieved 96% precision with 70% recall, and 96% precision with 82% recall, respectively, while SaRAD system [27] reported 95% precision with 85% recall. More recently, Sohn et al. [28] used a LF to SF matching algorithm similar to Yu et al. [25] and reported 96.5% precision with 83.2% recall. However, these performance measures are hardly comparable because each system was tested on different corpora [29]. Although both Chang et al. [23] and Schwartz et al. [26] used the Medstract Gold Standard Evaluation Corpus [30], each had made undisclosed modifications to their test corpus [29], resulting in difficulty in comparison. Nevertheless, Torii et al. [31] performed a meta-study to compare the results of a number of AR systems and found that the SF-LF identified by each system is generally consistent with previous reports. In general, these systems can achieve excellent precisions but still have plenty of room for improvement in terms of recall. Currently, Schwartz et al. [26] and Sohn et al. [28] demonstrated the best AR performance than other existing systems.

Schwartz et al. [26] used a 2-step algorithm for AR under the assumption that the SF-LF must exist in the same sentence. In the first step, identification of a possible SF-LF pair is initiated by the presence of a pair of brackets. It considered two cases - the LF is in the brackets or the SF is in the brackets. If it is likely that the SF is in the brackets, the second step is to search for the LF word boundaries in the sentence by morphological features. Sohn et al. also used brackets to initiate the process of AR but ignored a list of common bracket-delimited structures, such as "(p < 0.05)". This is followed by filtering the potential SF-LF pairs using a set of pre-defined rules.

Our approach to AR is based on machine-learning and exploits a novel set of rich features to describe properties of a potential SF-LF pair. In addition, the difference between our system and those of [26,28] is that we can identify pairs with unused characters in the SF. For example, "CA5" and "CA V gene". Our system also outputs the prediction probability to indicate the confidence of each identified SF-LF pair. Tested on the AB3P corpus [28], our system demonstrated a F-score of 89.90% with 95.86% precision at 84.64% recall. We also annotated a corpus of 1200 PubMed abstracts which was derived from BioCreative II gene normalization dataset. On our corpus, our system achieved F-score of 86.20% with 93.52% precision at 79.95% recall. Comparing to existing available AR systems [26,28], our system outperformed them on both corpora and performs about 14 times faster than the best AR performance system [28]. All resources can be found on our website. By applying our system to extract all short form-long form pairs from all available PubMed abstracts, we have constructed BIOADI, the most comprehensive dictionary of biological abbreviations online. Mining BIOADI reveals many interesting trends of bio-medical research.

## Methods

### Preparation of training data

We annotated a corpus of 1200 abstracts from BioCreative II gene normalization dataset [32] by a single person for consistency and exploited it to develop an AR system. Hence, we denote this annotated corpus as "BIOADI corpus." We followed the style and the annotation guideline of AB3P corpus [28], in which SF and LF pairs are separated by "|" (for example, "HSP" and "heat shock protein" form "HSP|heat shock protein") to annotate each abstract. We focus on the following forms of SF-LF pairs:

1. LF is in front of SF, and SF is in brackets or square brackets, *e.g. *"HSP (heat shock protein)";

2. SF is in front of LF, and LF is in brackets or square brackets, *e.g. *"heat shock protein (HSP)";

3. Both SF and LF are in brackets or square brackets and separated by comma or semi-colon, *e.g.*" (HSP, heat shock protein)".

The SF-LF pairs adhered to one of these forms will be annotated as potential SF-LF pairs. The BIOADI corpus includes 1668 true SF-LF pairs and 145 synonym pairs which are marked with "//" in the beginning of each pair. The synonym pairs were not considered as valid SF-LF pairs and ignored in the following experiments. Meanwhile, We also used the AB3P corpus for performance evaluation. It contains 1221 true SF-LF pairs. Some of them are synonym pairs, however.

Both positive and negative instances were required for model training. In this study, annotated SF-LF pairs were used as positive instances in training data, and negative instances were automatically extracted from text. The extraction of potential SF-LF pairs was similar to the previous work [28]. However, constraints on character lengths or word lengths of SFs were not set, but numbered list indicators (*e.g.*, (a), (b), (1a), (1b), (I), (II)....) and common strings ("e.g.", "and"...) were filtered out. Potential SFs which do not contain any alphabetic character or contain certain symbols ("=", "%", ">" and "<") were excluded. A potential LF can be composed of up to ten consecutive words preceding a potential SF in the same sentence, or in brackets or in square brackets following a potential SF which means that there are at most ten potential LFs of a potential SF, of which one of them is correct. Each abstract was split into sentences by "sentence and paragraph breaker" [33] before the automatic AR process. All potential SF-LF pairs were checked for existence in the list of positive instances. If not, the pairs acted as negative instances in training data.

### Feature extraction of SF-LF pairs

Before training and testing the model, it is a pre-requisite applying to transform the pair into the form of a feature vector. In order to construct features from raw data (potential SF-LF pairs extracted from the previous step), we defined four sets of features. The design of these features was originated from [16], inspired by the previous works [29,34] and carefully selected in our tests. The detail is as the following:

• String morphological features of SF and LF

We had selected the following binary features to describe the string morphology in order to extract and represent the literal information and character properties of each SF and LF. We had also used some features to demonstrate the position and amount of stop words in LFs. For example,

1. Is the first letter of the string uppercase?

2. Is the first letter of the string lowercase?

3. Are all characters of the string all uppercase?

4. Are all characters of the string all lowercase?

5. Does the first word of the LF use the first letter of the SF (case-sensitive and insensitive)?

6. Is the first word of the LF a stop word (case-insensitive)?

7. Is the first word of the SF a stop word (case-sensitive)?

8. Does the string contain numbers?

9. Does the LF share the same numbers of the SF?

10. Does the string contain Greek alphabet?

11. Does the LF start with the SF?

12. Does the brackets or square brackets of the string pair well?

We also applied the discrete binary features to characterize the composition of each SF and LF, including:

1. Is the number of stop words in the LF = 1, 2, 3, 4...?

2. Is the length (in tokens) of the string = 1, 2, 3, 4...?

3. Does the string contain certain punctuation symbol?

4. The character pattern of the SF: First, to convert each consecutive uppercase or lowercase characters to "A" or "a" depending on whether they are uppercase or lowercase. Second, to convert each consecutive digits to "1". Third, to prune off other characters. This is followed by matching the converted string to a specified pattern. For example, the SF "Rb1" matches the pattern "Aa1".

• LF tokens

We used space and punctuations as delimiters to tokenize each potential LF into tokens. Each token acted as a binary feature to represent token information of the potential LF. We also applied token bi-grams as binary contextual features of the potential LF.

• Numeric features between SF and LF

We exploited this set of features to describe the mapping of SF letters to LF letters and the calculation of the character usage between SF and LF.

1. The number of characters of longest common subsequence of the SF-LF pair divided by the SF length (in characters) [35];

2. Same as 1 but with the string consisting of the first character of all LF tokens (*e.g. *"protein kinase C" forms "PKC");

3. The size of sharing character set between the SF and the LF divided by the size of character set of the SF;

4. The size of character set of the SF divided by the SF length (in characters);

5. The shortest LF of the SF-LF pair extracted by Schwartz's AR system [26] that is equal to the LF;

6. Same as 5 but ignoring numbers of the SF and LF (*e.g. *"CA 5 gene" are transformed into "CA gene");

7. Same as 5 but reversing both the SF and LF (*e.g. *"CA 5 gene" are transformed into "eneg 5 AC");

8. Same as 7 but ignoring numbers of the SF and LF (*e.g. *"CA 5 gene" are transformed into "eneg AC");

• Contextual features of SF-LF pair

We generated contextual information of each potential SF-LF pair from the tokens which precede the SF-LF pair and are limited two tokens at most. Those tokens acted as binary features respectively.

We also applied token bi-grams as binary contextual features of the SF-LF pair.

The total number of each of set of features and the total number of all features are listed in Table 1.

**Table 1:** Number of features of each feature set and total number of all features generated in feature extraction

Feature Set(s)	BIOADI corpus	AB3P corpus
M	251	239
L	23601	25993
N	8	8
C	19264	20231

M + L + N + C	43124	46471

M, String morphological features; L, LF tokens; N, Numeric features; C, Contextual features

### Model training and testing

To test the performance of different learning algorithms in our feature set, we implemented four learning algorithms, including Support Vector Machine, Naïve Bayes, Logistic Regression and Monte-Carlo Sampling Logistic Regression. We took advantage of MALLET [36] to implement Naïve Bayes, Logistic Regression and Monte-Carlo Sampling Logistic Regression and LIBSVM [37] for SVM. In this study, LIBSVM was incorporated into MALLET to simplify the pipeline of experiments on various learning algorithms.

We also set a ruled-based filter in the post-processing step to clean up some easily fixed mistakes to improve the precision. The output SF-LF pairs were filtered by the following rules generalized from the inside tests:

1. If the length of the SF (in characters) is equal to one, the length of the LF (in words) must not be large than one;

2. If the SF is equal to "s", the first letter of the LF must not be "S" or "s" (*e.g. *"substract(s)");

3. The brackets and parentheses of the SF and LF must pair well;

4. The LF cannot contain a semi-colon followed by a space;

5. The number of punctuations in the SF normalized by the length of the SF (in characters) must not be large than 0.5;

6. The pairs of bracket or parenthesis are at most two pairs;

7. The LF must not start with the SF;

8. The SF must not be a sequence or list indicator (*e.g.*, (a), (b), (1a), (1b), (I), (II)....);

Since Sohn's and Schwartz's AR systems are available online, we were able to reproduce their systems at our local site. Generally speaking, we used them without any modification in the whole process of system evaluation and comparison. We only made a necessary modification in the part of input and output of Schwartz's system for handling the format style of the AB3P corpus.

## Results and discussion

In this study, we used a machine learning approach to SF-LF pair recognition instead of a rule-based approach [26-28,38]. Four learning algorithms, Logistic Regression, Monte-Carlo Sampling Maximum Entropy, Support Vector Machine and Naïve Bayes, were tested.

### Learning algorithms and feature sets analysis

We evaluated the performance of the learning algorithms on both corpora (BIOADI and AB3P, as described in Methods). The performance of each algorithm was carried out using one corpus for model training and the other for model testing, vice versa. As tabulated in Table 2, our results showed that the F-scores of different learning algorithms tested on the BIOADI corpus were between 64.54% and 86.22%. The performances on AB3P corpus were between 85.03% and 89.90%. The F-score difference among the learning algorithms trained using the AB3P corpus was larger than using the BIOADI corpus suggesting that pairs of SF and LF were more irregular in the AB3P corpus (containing synonyms) than in the BIOADI corpus. Our result also indicates that logistic regression and support vector machine with RBF kernel outperformed other algorithms in both precision and recall on both corpora with our feature set. As the precision of logistic regression being higher than SVM with RBF kernel, the logistic regression algorithm was used to develop our AR system.

**Table 2:** Performance of various learning algorithms tested on the BIOADI corpus and the AB3P corpus

Training Corpus	AB3P corpus			BIOADI corpus		
Test Corpus	BIOADI corpus			AB3P corpus		
Learning Algorithm	*Precision*	*Recall*	*F-score*	*Precision*	*Recall*	*F-Score*
Naïve Bayes	0.9733	0.4828	0.6454	0.9784	0.7518	0.8503
Logistic Regression	0.9352	0.7995	0.8620	0.9586	0.8464	0.8990
MCMaximun Entropy	0.9320	0.7013	0.8004	0.9301	0.8066	0.8640
SVM (linear kernel)	0.9446	0.7808	0.8549	0.9619	0.8398	0.8967
SVM (RBF kernel)	0.9212	0.8103	0.8622	0.9256	0.8580	0.8906

We evaluated the four sets of features on the two corpora with logistic regression. Table 3 presents the performance of four trials on different combinations of four sets of features on both corpora. The F-scores of these trails range from 80.87% to 85.81% for the BIOADI corpus and range from 86.88% to 89.90% for the AB3P corpus. Comparing the trials with the highest F-score with the lowest one on both corpora, the trails with all features were four to five percent higher than the one with only morphological set of features. They also performed the best in both precision and recall on both corpora. That suggests our feature set is robust and reliable.

**Table 3:** Performance of logistic regression classifier trained with different feature sets and tested on the BIOADI corpus and the AB3P corpus

Training Corpus	AB3P corpus			BIOADI corpus		
Test Corpus	BIOADI corpus			AB3P corpus		
Feature Set(s)	*Precision*	*Recall*	*F-score*	*Precision*	*Recall*	*F-Score*
M	0.9155	0.7242	0.8087	0.9392	0.8082	0.8688
M + L	0.9153	0.7489	0.8238	0.9401	0.8207	0.8763
M + L + N	0.9260	0.7995	0.8581	0.9556	0.8398	0.8939
M + L + N + C	0.9352	0.7995	0.8620	0.9586	0.8464	0.8990

M, String morphological features; L, LF tokens; N, Numeric features; C, Contextual features

### Comparison with previous works

We compared our system to Schwartz's and Sohn's systems. Each system was trained with the AB3P corpus before tested them with the BIOADI corpus and vise versa. Medstract Gold Standard Evaluation Corpus for evaluation [30] was not used as past results with the corpus reported are all based on the different modification version annotated by each team [29].

The results are shown in Table 4. The F-scores of the systems on the BIOADI corpus were between 85.12% and 86.20%, while that on AB3P corpus were between 86.13% and 89.90%. The highest precision on both corpora were achieved by Sohn's system, but the highest F-score and the highest recall on both corpora were achieved by our system. The F-score of our system was three percent higher than Schwartz's system on the AB3P corpus. These results suggested that our system outperformed Schwartz's and Sohn's systems.

**Table 4:** Performance of the AR systems tested on the BIOADI corpus and the AB3P corpus

Training Corpus	AB3P corpus			BIOADI corpus		
Test Corpus	BIOADI corpus			AB3P corpus		
System	*Precision*	*Recall*	*F-score*	*Precision*	*Recall*	*F-Score*
This study	0.9352	0.7995	0.8620	0.9586	0.8464	0.8990
Sohn et al. [28]	0.9482	0.7832	0.8578	0.9701	0.8356	0.8979
Schwartz et al. [26]	0.9416	0.7766	0.8512	0.9500	0.7883	0.8613

We performed a significant test to measure the confidence of our results. The null hypothesis is that our system and another system performs equally well. Applying the bootstrap test, we tested one system against the another for F-score on both corpora with 1000 repetitions. The results are shown in Table 5. Compared against Sohn's system, our system won 810 in 1000 times of bootstrap tests on the BIOADI corpus, and won 619 times on the AB3P corpus. The p-values was less than 0.001 in both conditions; thus, rejecting the null hypothesis. Comparing against Schwartz's system, our system won 989 in 1000 times of bootstrap tests on the BIOADI corpus, and won 1000 times on the AB3P corpus. The p-values was less than 0.001, rejecting the null hypothesis. Hence, our results suggested that our system is an statistically significant improvement over Schwartz's and Sohn's systems.

**Table 5:** Significant tests among the AR systems on the BIOADI corpus and the AB3P corpus

	BIOADI corpus			AB3P corpus		
Comparing with	*Sampling*	*Won*	*P-value*	*Sampling*	*Won*	*P-value*
Sohn et al. [28]	1000	810	< .0001	1000	619	< .0001
Schwartz et al. [26]	1000	989	< .0001	1000	1000	< .0001

### Learning curve analysis

**Figure 1:**
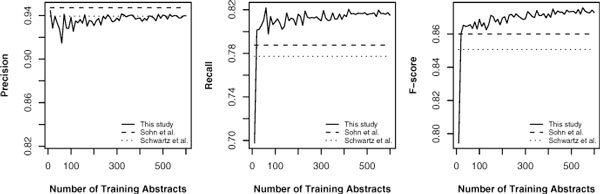
**Performance versus training data size tested on the BIOADI corpus**.

**Figure 2:**
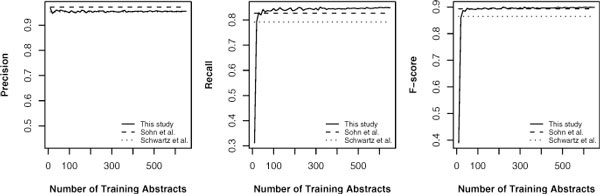
**Performance versus training data size tested on the AB3P corpus**.

**Figure 3:**
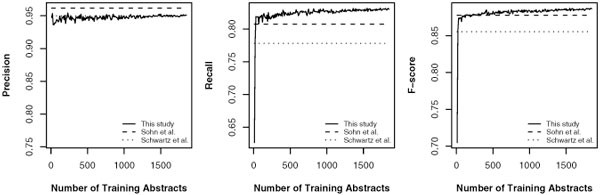
**Performance versus training data size tested on the merged corpus (AB3P corpus + BIOADI corpus)**.

We were interested in the influence of training data size to the performance. We randomly selected 600 abstracts as test data from each corpus, with the rest of the corpus as training data. The models were trained using 10 randomly selected abstracts at the first iteration and increased the training data size by 10 randomly selected abstracts at each iteration. The system performance of each iteration were recorded and averaged after five repeated tests. The five tests used five different test data which consisted of 600 randomly selected abstracts. Schwartz's and Sohn's systems were tested as the control groups in the experiment. Figure 1, 2 and 3 shows the results on the two corpora and merged corpus (BIOADI corpus + AB3P corpus).

Figure 1 shows the results of three AR systems tested on the BIOADI corpus. In the figure of precision versus the training data size, at first the precision is below the lines of Schwartz's and Sohn's systems and fluctuates intensely. However, the range of the fluctuation decreases as the size increasing and the curve gets close to the line of the precision of Schwartz's system at 94%. There is not much difference of precision among these systems. In the figure of recall versus training data size, the recall of our system is higher than other systems' recall even when the system was trained by a small size of training data. The range of oscillation also decreases when the size of training data increases. The recall in the last iteration (trained by 600 abstracts) was three percent higher than Sohn's system and four percent higher than Schwartz's system. We observed the same trend in the figure of F-score versus the size of training data. At first, the performance was not much different among the systems. After using more training data, our system shows the advantage of machine learning based AR system by improving its performance.

Figure 2 shows the same experiment but using the AB3P corpus for testing. In this case, our system also performed better in recall and F-score. Unexpectedly, in this case, a difference between the results obtained from the corpus is that our precision was better than Schwartz's system. It indicates that training with a consistently annotated corpus (i.e., BIOADI corpus) is useful to improve AR performance.

In addition, to test the influence of data size in a large set of training data, we merged both corpora to form a new dataset which contains 2450 unique abstracts. Figure 3 shows that the trends in precision, recall and F-score were similar to the results in Figure 1 and Figure 2. As the data size increases, it is expected that our machine learning-based approach will continue to improve while no improvement will be observed for rule-based systems.

### Computational cost analysis

Due to the rapid increase of biomedical articles, the throughput of an AR system is important for dealing with large quantities of articles. Hence, we were interested in comparing the processing speed of three systems. The test platform is on a computer with Intel Core Quad CPU 2.4 GHz, 5 gigabytes of RAM and 32bit Linux system. The test time is defined as the elapsed CPU time between program invocation and termination. We tested the systems on four data sizes (1200, 1250, 2450 and 5000 PubMed abstracts with 240918, 229501, 470419 and 988828 tokens, respectively). The test results are shown in Table 6. The fastest system on all corpus size was achieved by Schwartz's system. It only took 3.318 seconds to deal with 5000 abstracts. Although our system took 45.506 seconds to deal with that size of abstracts, it was more than 14 times as fast as Sohn's system, which took 630.917 seconds to process the data. On a larger scale, 17,551,169 PubMed abstracts stored in our local database were processed. Our system completed the whole process in 15 hours, suggesting that the efficiency of our system is acceptable to deal with large quantities of text for other text-mining applications.

**Table 6:** Testing time (in seconds) of three AR systems testing on different size of PubMed abstracts

	Testing Size of Abstracts (Tokens)			
System	1200 (240918)	1250 (229501)	2450 (470419)	5000 (988828)
This study	13.355	13.316	25.059	45.506
Sohn et al. [28]	159.292	135.254	292.343	630.917
Schwartz et al. [26]	0.873	0.897	1.598	3.138

### Analysis of mis-identification cases

In the analysis of false-positive (FP) pairs produced by our system, the common FP pairs are due to partial match which has also been reported by previous works [26,28]. For example,

• "PPIs|pump inhibitors" rather than "PPIs|proton pump inhibitors"

• "CCR5|chemokine receptor 5" rather than "CCR5|C-C chemokine receptor 5"

Other common types of the pairs missed by our system include out of order match (*e.g.*, NGL-1|netrin-G1 ligand) and partial match (*e.g.*, Pol II|RNA polymerase II). There is another type of false-negative pairs reported [26,28], which are with unused characters in the SF. Most of them has been removed in the process of AR for false-positive reduction. However, we kept them and identified them with model prediction correctly. For example, our system can identify:

• "CA5|CA V gene"

• "FTH1|ferritin heavy-chain gene"

### BIOADI

To construct a comprehensive biological abbreviation dictionary, we identified SF-LF pairs from 17,551,165 PubMed abstracts. The final AR system for this purpose is trained by the BIOADI corpus. Therefore, we expect that its F-score can reach about 90%. We did not include the AB3P corpus because it contains some inconsistent synonyms, as mentioned earlier. A total of 8,306,789 SF-LF pairs were identified. These SF-LF pairs are grouped as 1,687,063 unique SF-LF pairs. Most of the SFs are 3 to 6 character long. Table 7 lists the top 20 most common abbreviations used in the scientific community. Although it is not possible to draw any conclusions with regards to research trends by an analysis of common abbreviations, a number of interesting observations can be made. Firstly, HIV (human immunodeficiency virus) and HCV (hepatitis C virus) are the only 2 viruses on the list. HCV/HIV co-infection is known to be a serious medical condition and there are a number of journals (such as Journal of Acquired Immunodeficiency Syndrome) devoted to HIV research. In addition, ELISA is a common serological technique for detecting anti-viral antibodies suggesting viral infection, including that of HIV. Secondly, CT and MRI scans are commonly used medical procedures in the diagnosis of AD (Alzhemier's disease) and RA (rheumatoid arthritis). Clinically, BMI (body mass index) and BP (blood pressure) are important physiological parameters linked to obesity and can be easily measured. Odds ratio (OR) is a commonly reported statistical parameter in epidemiology and since medical conditions appears to dominate this list, it is not surprising to find OR here as well. PCR (polymerase chain reaction) remains a useful molecular technique in many areas of research and it is no wonder that Professors Kary Mullis and Michael Smith were awarded the 1993 Nobel Prize in Chemistry for its development. Confidence intervals (CI) are reported in nearly all statistical measure; hence, it is not surprising to be grabbing the top seat in this list.

**Table 7:** Top 20 most frequent SF-LF pairs extracted from 17,551,165 PubMed abstracts

Rank	Short Form	Long Form	Frequency	Class
1	CI	confidence interval	36142	Statistical Measure
2	PCR	polymerase chain reaction	26951	Experimental Technique
3	NO	nitric oxide	25229	Medical Relevance
4	CT	computed tomography	20084	Experimental Technique
5	HIV	human immunodeficiency virus	20027	Virus Studied
6	LPS	lipopolysaccharide	20027	Experimental Technique
7	OR	odds ratio	19914	Statistical Measure
8	MRI	magnetic resonance imaging	19396	Experimental Technique
9	IL	interleukin	16934	Medical Relevance
10	CNS	central nervous system	15915	Medical Relevance
11	BMI	body mass index	15614	Clinical Observation
12	RA	rheumatoid arthritis	14221	Medical Relevance
13	AD	Alzheimer's disease	14103	Medical Relevance
14	PKC	protein kinase C	13958	Medical Relevance
15	ELISA	enzyme-linked immunosorbent assay	13946	Experimental Technique
16	CSF	cerebrospinal fluid	13815	Medical Relevance
17	DA	dopamine	11297	Medical Relevance
18	BP	blood pressure	10991	Clinical Observation
19	MR	magnetic resonance	10973	Experimental Technique
20	HCV	hepatitis C virus	10944	Virus Studied

One of the complexity of SF and LF is that a single SF can have multiple LF, depending on context. For example, APC can have many different meanings, as illustrated in Table 8. It may be interesting to note that 3 of the top 5 are related to cell cycle control (adenomatous polyposis coli; anaphase-promoting complex) which are known to be relevant in cancer research and colon cancer (adenomatous polyposis coli; argon plasma coagulation). Antigen-presenting cells is widely known to be an important key to acquire immunity. Activated protein C is an important component in blood clotting pathway and may be interacting with warfarin, a widely used drug to manage deep-vein thrombosis and widely known for its extensive interactions with other medical drugs. Hence, it is not surprising to see a large occurrence of these terms in the literature. However, it also suggests that reading biomedical literature requires a good understanding of these terms in order to prevent ambiguity. Despite the contextual complexity, the extracted LF-SF pairs may be used to support future research, such as the development of named entity recognition systems or abbreviation disambiguation.

**Table 8:** Top 5 long-form occurrences for the abbreviation "APC"

Long Form	Frequency
antigen-presenting cells	2077
adenomatous polyposis coli	1108
activated protein C	924
anaphase-promoting complex	254
argon plasma coagulation	152

Our web site http://bioagent.iis.sinica.edu.tw/BIOADI/ provides freely access to two online services and two off-line tools. Online services include (1) SF-LF Search Service and (2) SF-LF Identification Service, whereas off-line tools include (1) an off-line abbreviation recognition tool and (2) an abstract fetching script. SF-LF Search Service helps users to quickly retrieve all of the SF-LF pairs in our database. Query results are listed as 20 records per page and ordered by the number of PubMed IDs of each pairs so that users can easily find out the most popular ones. To see in which PubMed IDs the SF-LF pair can be found, users can click on the document picture under the "PubMed" column to generate a "PubMed ID box." For those SF-LF pairs having too many PubMed IDs to be fully displayed in the box, users can click on the "PubMed Resource" to see the whole list of PubMed IDs. By using the search service, users can find different subtypes of SFs or LFs and thereby come upon extra PubMed IDs that they can not find through regular literature search. Secondly, "SF-LF Identification service" provides real-time AR service. In the identification service section, users can use the text area for text inputs such as abstracts or manuscripts, whereas another input box is for PubMed ID. After receiving the submission of inputs, the system will return identified SF-LF pairs and scores for each pairs. The higher the score is, the better the identification can be trusted. If the input is a PubMed ID, the result table will also show a hyperlink to PubMed at the bottom. In the download section, we provide all of the SF-LF pairs in our database with two helpful tools: (1) Off-line Abbreviation Recognition Tool and (2) Abstract Fetching Script. The off-line abbreviation recognition tool can automatically do AR on a given text file and generate SF-LF pairs and their score to the output file. Since it is a JAVA application, it can run on any platform and serve as a SF-LF identification component in any pipeline of analysis. The abstract fetching script is a Perl script and can be used to massively download abstracts through the Web Service of PubMed database for given PubMed IDs. To ensure the stability of our web site, all scripts and layout of the web site have passed tests on different browsers, different platforms, and even mobile devices.

## Conclusion

Our system demonstrated 93.5% precision and 80.0% recall, giving a F-score of 86.2%, which statistically significantly outperformed the existing best performance AR system. At the same time, our system runs sufficiently fast to handle the entire set of PubMed abstracts. This suggests that a machine learning approach to abbreviation recognition gives not only good performance as good as a rule-based system, but also satisfying execution.

## Competing interests

The authors declare that they have no competing interests.

## Authors' contributions

Cheng-Ju Kuo and Maurice HT Ling developed methods, annotated the corpus, implemented the offline software, and drafted the manuscript. Kuan-Ting Lin developed the online interface and revising the manuscript. Chun-Nan Hsu was responsible for all aspects of the project and helped revise the manuscript.

## Note

Other papers from the meeting have been published as part of *BMC Genomics* Volume 10 Supplement 3, 2009: Eighth International Conference on Bioinformatics (InCoB2009): Computational Biology, available online at http://www.biomedcentral.com/1471-2164/10?issue=S3.
